# Health conditions among farmworkers in the Southwest: An analysis of the National Agricultural Workers Survey

**DOI:** 10.3389/fpubh.2022.962085

**Published:** 2022-11-03

**Authors:** Sheila Soto, Aaron Meck Yoder, Tomas Nuño, Benjamin Aceves, Refugio Sepulveda, Cecilia Ballesteros Rosales

**Affiliations:** ^1^Division of Public Health Practice & Translational Research, Mel and Enid Zuckerman College of Public Health, University of Arizona, Phoenix, AZ, United States; ^2^Department of Epidemiology & Biostatistics, Mel and Enid Zuckerman College of Public Health, University of Arizona, Tucson, AZ, United States; ^3^School of Public Health, San Diego State University, San Diego, CA, United States

**Keywords:** National Agricultural Workers Survey (NAWS), farmworker, Southwest, Latinx, diabetes

## Abstract

Agricultural jobs pose many challenges to the health and wellbeing of a disadvantaged population. In the Southwest region, the socioeconomic factors of living along the U.S.-Mexico border, migration patterns, lack of access to health care, low utilization of health care services, lack of health insurance, and highly demanding occupation may uniquely affect health outcomes for farmworkers. This paper presents descriptive information for professionals to improve access to care by tackling barriers afforded by the agricultural industry. The National Agricultural Worker Survey (NAWS) is an employment-based, random-sample survey of U.S. agricultural workers in six regions: East, Southeast, Midwest, Southwest, Northwest, and California. We examined farmworkers' self-reported health conditions, including asthma, diabetes, high blood pressure, other chronic conditions, or any condition by region from 2013 to 2016. We used logistic regression to determine differences in lifetime prevalence of health conditions between farmworkers in the Southwest region (*n* = 727) and farmworkers in other regions (*n* = 8,850) using weighted data. After adjusting for age, gender, income, insurance status, and English-speaking ability, the odds of high blood pressure and other condition were similar in all regions. The prevalence of diabetes was almost double in the Southwest (114.2 per 1,000 farmworkers). The odds of diabetes were 1.31 (95% CI 0.99, 1.74) times greater in the Southwest region than in the other regions. Asthma was the only condition that was lower in the Southwest (22 per 1,000 farmworkers) compared to the other regions. The odds of asthma were 0.61 (95% CI 0.36, 1.03) times lower in the Southwest region than in other regions. The results follow previous studies on the prevalence of asthma among the farmworker population and elevated probability of chronic diseases including diabetes among the Latino population in the U.S.

## Introduction

Farmworkers play an essential role in the food chain yet work long hours, receive unreasonably low wages, no benefits, and are at a heightened risk of environmental and occupational exposures (1). Agricultural jobs are typically physically high-demanding with odd positioning and repetitive movement (1). Often, employers provide little support and harsh working conditions leading to reduced worker safety, greater musculoskeletal and respiratory health issues, and, in some cases, abusive supervisors (1–3). Potential adverse health exposures and outcomes include pesticide, heat, and sun exposure; injuries due to dangerous tools and machinery; infectious disease complications; musculoskeletal injuries; respiratory illness; mixed increased risk for chronic disease; chronic respiratory problems; cancer; depression; tuberculosis; neurological deficits; and higher rates of infertility and miscarriages (4, 5).

Chronic disease are the leading cause of death and disability in the United States (6, 7). Currently in the U.S., over 37.3 million adults are living with type 2 diabetes (T2D) and 96 million are prediabetic even though 8 of out 10 are unaware of their condition (8). T2D takes a staggering toll on individuals, families, and communities. While T2D can be found among all populations, there are significant disparities between ethnic groups (9). Racial and ethnic health disparities have shown that T2D is a social disease that has more to do with social inequities between groups rather than genetic factors (6, 10). For instance, social differences between non-whites and whites include social standing and earnings (6).

It is estimated that the rate of diagnosed diabetes is 50% higher among Latinos than non-Latino white adults (11). Estimates from the 2019 U.S. American Community Survey found that more than 60,000,000 U.S. Americans identify as either Hispanic or Latino (12). As the largest ethnic minority group, it is essential to note that Latinos in the U.S. have a higher proportion of T2D than any other racial group (13). Furthermore, Latinos have higher rates of diabetes complications including kidney failure and retinopathy than non-Latino whites (11). Individuals living in rural and unincorporated areas also experience an even greater disproportionate burden of diabetes and associated comorbidities when compared to individuals residing in urban areas (11). Similar to the U.S., chronic diseases in Mexico are also the leading causes of death and disability for the country (6, 7). Geographically, the border separating the two countries has been argued to be the poorest region of the U.S. due to the lag in economic development (6, 14). For years, the U.S.-Mexico border region's epidemiological profile includes high rates of cardiovascular disease, obesity, and diabetes (15). For instance, the rate of diabetes and obesity are amongst the highest in the world and most prominent among the Mexican American community (6). There are ~7.7 million people living along the southern U.S. border and another 6.7 million along Mexico's northern border (16). The area includes 48 U.S. counties in Texas, New Mexico, Arizona, and California; 80 Mexico municipalities in Tamaulipas, Nuevo León, Coahuila, Chihuahua, Sonora, and Baja California; along with 17 sister cities (15). The majority of the people living in the border region are people of color: 50% Hispanic or Latino, 4% Black or African American, and 10% Asian American or Pacific Islander, among others (17). About 33% of the population along the border is white compared to the overall U.S. average of 60% (17). Border residents are nearly twice as likely to be immigrants compared to the U.S. average, and about 23% of border residents were born in another country compared to 14% of the U.S. total (17). In the border region, about half of all immigrants are U.S. citizens (17). Border states are home to immigrants from many countries but more than 2.3 million immigrants have a Mexico-origin (17). Along the border region, unemployment rate is higher than the U.S. average with the highest being in Arizona (17). Texans on the border face almost twice the poverty rate compared to the U.S. average followed by New Mexico and Arizona.

Additionally, about half a million people cross the U.S.-Mexico border everyday (17). In 2021, the largest average daily crossings were in Texas, followed by California, Arizona, and New Mexico (17). These border states depend on farmworkers who commute daily by foot from Mexico to the farming site in the U.S. In Yuma County, Arizona, approximately between 8,000 and 10,000 workers cross the border daily during November to April to reach the farming site (18). Farmworkers living and working in the border region of the U.S. and Mexico have different health status than other regions due to the health disparities and socioeconomic factors of the border region including poor housing and work conditions (19). In the U.S., much of the farmworker housing have been linked to negative mental and physical health outcomes due to crowding, mold, mildew, and other allergens, pesticides, and structural deficiencies (19). Even though policy set by the Migrant and Seasonal Agricultural Worker Protection Act of 1983 is meant to set housing standards, historically it has not been enforced (19). Health care utilization among farmworkers and Latinos is low, meanwhile, about half made an ambulatory care visit in the previous year compared to 75.5% of non-Latino whites (20). Approximately half of the immigrant population are unauthorized to work in the U.S. further affecting their use of needed services (20). Utilization of preventative health services is also lower for farmworkers than both U.S. Latinos and non-Latino whites (20). Commonly, U.S. farmworkers lack medical insurance, and for those who do obtain insurance, they often do not know how to use it (20). Farmworkers may also lack transportation making access to any resources difficult (20). Additionally, each year up to 200,000 migrants travel from southern Mexico to the northwest region of country for agricultural work (21). In Mexico, migrant farmworkers are one of the poorest and more marginalized social groups within the country (21). Mexican farmworkers often suffer from malnutrition, food insecurity as well as harsh living and labor conditions (21). Similar to the U.S., previous research in Meixco highlights the health risks endured by workers and the precarious socioeconomic conditions such as low wages, low formal education, dietary changes due to migration, lack of access to health services, and high risk of obesity due to greater consumption of foods of low nutritional value (21).

Among children in the U.S., asthma is one of the most prevalent childhood illness affecting more than 10 million children under 18 years of age (22). Researchers have associated asthma with racial and economic inequities (22, 24). Under current federal laws, agricultural is one of the industries that allow children to work at a far younger age than children working in non-agricultural jobs (25). Despite continuously being labeled as the most dangerous industry in the U.S (22), agriculture accounts for 42% of all youth work-related deaths (25). Few studies have assessed respiratory health and asthma among children in agriculture. However, some research shows that hired Latino children farmworkers had two or more breathing problems during a study by von Glascoe and Schwartz (2019), and over 36% had suspected asthma with nearly 20% suffering from wheezing (25, 26). In other areas such as the San Joaquin Valley in California, childhood asthma rates can approach double the national rate (22, 26). In this Valley study, children of Mexican descent appear to have significantly higher asthma rates than their Mexican counterparts (22, 23, 26). Yet, conflicting research also shows that Mexican-born children under the age of 18 have lower lifetime asthma rates (ever diagnosed) and lower symptoms rates than other ethnic groups and Latino subgroup in the U.S (23). In adult farmworkers, respiratory illness and disease are common clinical diagnosed problems (25). The effects of acute or chronic exposure to organophosphorus pesticides can cause bronchial hyperreactivity and can trigger asthma attaches, exacerbate asthma, or increase the risk of developing asthma (22).

Some health outcomes are preventable or manageable with increased availability and affordability of health care services; improved continuity of care; and the use of culturally competent health strategies that consider linguistical differences and low health literacy (4). Along with these solutions, it must also be considered that most migrant and seasonal farmworkers (MSFWs) live in isolated rural areas that further complicate transportation to much needed resources and services (4). Farms are typically located in medically underserved areas that suffer from provider shortages and low-retention rates (4). Furthermore, the migratory movement of MSFWs make it challenging to obtain reliable health information, leading to an inaccurate denominator in morbidity and mortality calculations (5). To add to the disparities confronted by MSFWs, most of the literature is dated or result are often ungeneralizable to other regions. While there is limited peer-reviewed literature using federally-obtained randomized datasets, there are still limitation to the collection methods used by research teams and limited access to entire datasets making valuable information inaccessible. Thus, leaving gaps in the literature.

The purpose of this paper is to describe the secondary analysis of the National Agricultural Workers Survey (NAWS) data to determine possible differences in self-reported health outcomes for farmworkers in the Southwest region.

## Methods

The NAWS is a complex, multistage survey conducted by the U.S. Department of Labor, accounting for seasonal and regional changes in farmworker employment (27). The NAWS is an employment-based, random-sample survey of U.S. agricultural workers that collects data using face-to-face interviews. To be a participant, workers must be hired by an eligible establishment and completing an eligible task such as growing crops, plants, vines, trees and their seeds; primarily engaging in supplying labor; aerial dusting or spraying; cotton ginning; cultivating; farm management; planting; and vineyard cultivation. There are seven sampling levels: cycle, region, farm labor area (primary sampling unit), county, ZIP code, employer, and farmworker (27). Factoring cycles is necessary because agricultural work is seasonal and many farmworkers migrate with the season (27). The selection of regions and participant farms is dependent on the amount of farm labor in a region during a particular cycle (27). The database is divides the U.S. into six regions: East, Southeast, Midwest, Southwest, Northwest, and California (28). A map of the regions can be found at the National Agricultural Workers website: https://www.dol.gov/sites/dolgov/files/ETA/naws/pdfs/NAWS_6_Regions_Map.pdf. Farm expenditures, the Quarterly Census of Employment and Wages, employers commercial lists, and other similar sources determine the selection of counties, ZIP codes, and employers (27). Interviews with randomly selected farmworkers takes place at the worksite during break times, before, or after work and is conducted by the Office of Policy Development and Research by JBS International employees (27). The main purpose of the NAWS is to monitor the terms and conditions of agricultural employment and assess the conditions of farmworkers. For this paper, we analyzed NAWS data from 2013 to 2016 (*n* = 9,577).

We examined farmworkers' self-reported health conditions including “asthma,” “diabetes,” “high blood pressure,” “other conditions” (excluding the prior conditions), or the presence of one or more previously mentioned conditions as “any condition.” Farmworkers were assessed for conditions in response to the following, “Have you ever been told by a doctor or nurse that you have the following conditions: asthma, diabetes, etc.?” We determined the prevalence of asthma, diabetes, high blood pressure, other condition, or any condition using unweighted counts self-reported by farmworkers. Basic demographics include age, hourly wage, education level, English-speaking fluency, gender, and health insurance status. The calculation of counts and percentages using unweighted (raw) data and weights adjusted for sampling means and standard errors. We also share the counts and percentages of the country of birth and legal status of farmworkers in the Southwest and other regions using NAWS tables created by the DOL (29). Estimates for country of birth and legal status use unweighted (raw) data.

### Statistical analysis

We used logistic regression to adjust for the NAWS survey weights to account for complex sampling to determine differences in lifetime prevalence of health conditions between farmworkers in the Southwest region (*n* = 727) and farmworkers in other regions (*n* = 8,850) using data weighted for the sample design. A second logistic regression model fit the weighted data to adjust for NAWS survey weights and age, hourly wage, education level, English-speaking ability, gender, and health insurance status. A separate model fit the data for each health condition (asthma, high blood pressure, other condition, any condition)—unadjusted and adjusted models of odds ratios and 95% confidence intervals. Finally, a third model fit data with imputed values for observations with missing covariate data (*n* = 246). We then used a logistic regression model adjusted for age, hourly wage, education level, English-speaking ability, gender, and health insurance status to determine each health condition's odds ratio using data with imputed values. We used multiple imputations using hot deck multiple imputations to generate missing data values. Hot deck imputation is a method used for handling missing data by replacing missing values of one or more variables of a non-respondent with observed values of respondent of a similar characteristic (30).

## Results

### Demographic characteristics

Table 1 shows descriptive statistics for farmworkers in the Southwest and other regions. The average age of farmworkers in the Southwest was greater (41.9 years) than other regions (38.1 years). Farmworkers employed in the Southwest had a lower average hourly wage ($9.17) than other regions ($10.50). Farmworkers in the Southwest averaged 8 years of schooling, while farmworkers in other regions averaged slightly more (8.4 years). The Southwest region hired more male (82.9%) workers who spoke English well (30.1%) compared to other regions (76.8 and 25.7%, respectively). Farmworkers in all other regions were more likely to have health insurance (39.7%) than farmworkers in the Southwest (33.8%).

**Table 1:** Demographic characteristics of farmworkers interviewed in NAWS, Southwest compared to other regions 2013–2016.

	**All regions *n* = 9,577**	**Southwest only *n* = 727**	**Regions other than Southwest *n* = 8,850**
Age, mean (SE)^a^	38.3 (0.31)	41.9 (0.98)	38.1 (0.33)
Hourly wage, mean (SE)	$10.40 (0.06)	$9.17 (0.12)	$10.50 (0.06)
Highest grade completed, mean (SE)	8.4 (0.09)	8.0 (0.28)	8.4 (0.10)
**English speaking ability, (%)**
Not at all	2,669 (27.9)	197 (27.1)	2,472 (27.9)
A little	3,259 (34.0)	225 (31.0)	3,034 (34.3)
Somewhat	1,117 (11.7)	85 (11.7)	1,032 (11.7)
Well	2,494 (26.0)	219 (30.1)	2,275 (25.7)
No response	38 (0.4)	1 (0.1)	37 (0.4)
**Gender, (%)**
Male	7,396 (77.2)	603 (82.9)	6,793 (76.8)
Female	2,181 (22.8)	124 (17.1)	2,057 (23.2)
**Insurance status, (%)**
Has insurance	3,763 (39.3)	246 (33,8)	3,517 (39.7)
Doesn't have insurance	5,777 (60.3)	478 (65.7)	5,299 (59.9)
Don't know	34 (0.4)	3 (0.4)	31 (0.4)
No response	3 (0.0)	–	3 (0.0)

a Mean and standard error are weighted to adjust for sample design.

Table 2 shares estimates of the country of birth and legal status found on the NAWS site for farmworkers in the Southwest and other regions (27). Estimates are unweighted (raw) data and show that the Southwest has more foreign-born (75%) and less U.S.-born (26%) workers than other regions (68 and 32%, respectively). The Southwest also houses more authorized (66%) than unauthorized (34%) workers compared to other regions (56% and 44%). Further examination into authorized status show that there are more permanent residents in the Southwest region (34%) compared to other regions (15%).

**Table 2:** Place of birth & legal status of farmworkers interviewed in NAWS, Southwest compared to other regions 2013–2016^*a*^.

	**All regions *n* = 9,577**	**Southwest only *n* = 727**	**Regions other than Southwest *n* = 8,850**
U.S. born	0.26 (*n* = 2,490)	0.25 (*n* = 182)	0.32 (*n* = 2,867)
Foreign born	0.74 (*n* = 7,087)	0.75 (*n* = 545)	0.68 (*n* = 5,983)
**Country of birth**
Mexico	0.68 (*n* = 6,512)	0.73 (*n* = 531)	0.60 (*n* = 5,328)
Central America	0.05 (*n* = 479)	0.02 (*n* = 15)	0.05 (*n* = 460)
Other	0.01 (*n* = 96)	– (–)	0.01 (*n* = 80)
**Current legal status**
U.S. citizen	0.03 (*n* = 2,873)	0.31 (*n* = 225)	0.36 (*n* = 3,195)
Permanent resident	0.21 (*n* = 2,011)	0.34 (*n* = 247)	0.15 (*n* = 1,345)
Work authorized	0.01 (*n* = 96)	0.01 (*n* = 7)	0.05 (*n* = 416)
Authorized	0.52 (*n* = 4,980)	0.66 (*n* = 480)	0.56 (*n* = 4,991)
Unauthorized	0.48 (*n* = 4,597)	0.34 (*n* = 247)	0.44 (*n* = 3,859)

^a^Unweighted estimates should be interpreted with caution because they have relative standard errors between 31 and 50 percent.

### Reported health conditions

Prevalence and adjusted and unadjusted odds ratios compared 727 farmworkers in the Southwest region to 8,850 farmworkers in other regions (Table 3). The prevalence of asthma was lower for farmworkers in the Southwest region and the adjusted odds ratio was 0.65 (95% CI 0.65, 1.10). The prevalence of diabetes was 114.2 per 1,000 farmworkers in the Southwest compared to 61.9 among farmworkers in other regions, and the adjusted odds ratio was 1.31 (95% CI 0.99, 1.74). Farmworkers in the Southwest reported high blood pressure at a higher prevalence (114.2 per 1,000) than farmworkers in other regions (94.1 per 1,000). The adjusted odds ratio for high blood pressure among farmworkers in the Southwest was 0.95 (95% CI 0.72, 1.26) making it similar to other regions. The prevalence of other conditions (not including asthma, high blood pressure or diabetes) was 79.9 per 1,000 farmworkers in the Southwest region compared to 57.9 per 1,000 farmworkers in all other regions, and the adjusted odds ratio was 0.86 (95% CI 0.62, 1.22). Results from the sensitivity analysis (Table 3) were similar to the primary analysis, and the adjusted odds ratio and 95% confidence interval varied by less than 0.03 compared to the primary analysis.

**Table 3:** Health conditions reported by farmworkers interviewed in NAWS, Southwest (*n* = 727) compared to other regions (*n* = 8,850) 2013–2016.

	**Count**	**Prevalence (per 1,000 farmworkers)^a^**	**Unadjusted OR (95% CI)**	**Adjusted OR^b^ (95% CI)**	**Sensitivity analysis OR^c^ (95% CI)**
**Asthma**					
Other regions	288	32.5	1.00	1.00	1.00
Southwest	16	22.0	0.61 (0.36, 1.03)	0.65 (0.38, 1.10)	0.65 (0.38, 1.11)
**Diabetes**					
Other regions	548	61.9	1.00	1.00	1.00
Southwest	83	114.2	1.58 (1.22, 2.05)	1.31 (0.99, 1.74)	1.32 (0.99, 1.74)
**High blood pressure**					
Other regions	833	94.1	1.00	1.00	1.00
Southwest	83	114.2	1.12 (0.87, 1.45)	0.95 (0.72, 1.26)	0.93 (0.71, 1.23)
**Other condition^d^**					
Other regions	512	57.9	1.00	1.00	1.00
Southwest	58	79.9	0.90 (0.64, 1.25)	0.86 (0.62, 1.22)	0.85 (0.61, 1.20)
**Any condition**					
Other regions	1,932	218.3	1.00	1.00	1.00
Southwest	189	260.0	0.94 (0.78, 1.13)	0.83 (0.68, 1.01)	0.82 (0.67, 1.00)

a Prevalence not weighted to adjust for survey design.

b Adjusted for age, gender, hourly wage, insurance status, education, and English-speaking ability.

c Adjusted for age, gender, hourly wage, insurance status, education, and English-speaking ability, missing values (n = 246) imputed using multiple imputation.

d Other conditions is defined as: “Have you ever in your whole life been told by a doctor or nurse that you have the following conditions: OTHER?”

This paper included almost 10,000 participants from six regions and there was only 274 (2.8%) missing outcome or covariate data. The sensitivity analysis suggests it was not a large factor in the results.

## Discussion

The use of a 30-year old database, such as the NAWS, allows for a representative sample of farmworkers. After adjusting for age, gender, income, insurance status, and English-speaking ability, the odds of high blood pressure and other condition were similar among farmworkers in the Southwest and other regions. However, the prevalence of diabetes was almost twice as high among farmworkers employed in the Southwest region (114.2 per 1,000 farmworkers). The odds of diabetes were 1.31 (95% CI 0.99, 1.74) times greater among farmworkers in the Southwest region compared to the other regions. As previously mentioned, Latinos in the border region have reported higher levels of T2D than the rest of the United States and Mexico (19, 20). Some reasons include the socioeconomic factors associated with living along the southern border; unsafe work and housing conditions; strenuous migration patterns; barriers with restrictive immigration status; and barriers to access care that lead to the underutilization of health services (19, 20).

Results also show that asthma in the Southwest is less than other regions. While these findings follow some of the previous studies regarding low rates of asthma diagnoses among farmworkers (25, 26), it is not quite clear why there is a discrepancy among regions. However, considering that the Southwest hires more Mexican-born workers than other regions, researchers have found that asthma rates among Mexican-born children tend to be lower than children not born in Mexico and some researchers believe it may be due to possible genetic protective mechanisms (23, 24). The protective factors are created by the continuous exposure to environmental toxins in and around farmworkers' homes (22–24).

A limitation of the NAWS dataset is the variation of health conditions within each region. Obtaining access to data at the state and county level on farmworker health status could improve geographical variations and inform best practices to address the health conditions unique to each region. Having access to detailed information such as workers' access to medication, utilization of medical care, duration of health diseases/conditions, country of diagnosis, access to safe housing, food security, among other factors could help researchers and farmworker advocates improve conditions for this vulnerable population. Unfortunately, the restrictive accessibility to this data make it unfeasible. Additionally, NAWS collects data through self-reported interviews conducted on farms; however, the presence of others (such as supervisors or foremen) may influence survey responses. It is also important to consider that often farmworkers do not report any health conditions because they cannot afford the care to receive a comprehensive health assessment that could potentially lead to a diagnosis or fear retaliation of job loss if discovered having a health condition. Another limitation is the exclusion of H2A visa holders, a growing population in U.S. agriculture. While the guest worker programs have evolved throughout the years, it is crucial to include this population as they encounter very similar experiences as MSFWs. Lastly, there is limited recent literature available on farmworker health to help navigate the barriers experienced by farmworkers. Much of the literature is dated or focus on small farmworker populations at a moment in time making findings ungeneralizable to other areas.

A strength of the NAWS study is that it is the only survey in the U.S. that uses randomized sampling and on-site interviewing by region to estimate the number of MSFWs since 1989. The methodology and positionality of NAWS allows for data collection to occur on-site, an opportunity typically closed to researchers or agencies.

### Public health implications

Agricultural jobs pose many challenges to the health and wellbeing of a disadvantaged population (4, 5). In the Southwest region, socioeconomic and racial/ethnic factors associated with living in the U.S.-Mexico border region, farmworker migrant streams including the daily commute of two countries, and the high-demanding labor intensive occupation uniquely affect the health outcomes for farmworkers in this region. The results from this paper follow previous studies on the elevated probability of diabetes among Latinos in the Southwest. Results show that progress still needs to be made along the U.S.-Mexico border to address health disparities that affect continue to affect the Latino population. This paper also shows similar findings from previous studies on lower asthma prevalence among Mexican origin Latinos compared to other Latino subgroups and non-Latino whites. While there is no clear reason to this result, it is worth further investigation of genetic protective mechanisms among farmworkers in Southwest as the findings could also potentially offer insight to the other regions. Often, cultural beliefs, lack of access to health services, dangerous environments, or inability to afford health care exacerbate chronic diseases (15). Not to mention that the diagnosis of one disease is commonly accompanied by another disease diagnosis. The findings elicit potential research questions about immigrant health status before and after migration, social determinants of health, long-term environmental childhood exposures, cardiometabolic factors, gestational diabetes, the gut microbiome, stress and epigenomics, among others conditions.

The cost of health care, little to no medical coverage, and lack of labor benefits can contribute to the low reporting of health conditions among farmworkers in all regions as some may be unaware of their conditions. A recommendation would be for binational collaboration between the U.S. and Mexico to promote chronic disease management and prevention geared toward farmworkers near the border *via* existing partnerships between health departments, local organizations, Federally Qualified Health Centers (FQHCs), academic institutions, farmworker agencies, and agricultural workers. It is also critical to advocate for appropriate and safe policies that improve farmworker health and to hold employers accountable to housing and work policies in place.

## Data availability statement

The datasets presented in this study can be found in online repositories. The names of the repository/repositories and accession number(s) can be found below: https://www.dol.gov/agencies/eta/national-agricultural-workers-survey/data NIOSH and EPA Variables.

## Ethics statement

Ethical review and approval was not required for the study on human participants in accordance with the local legislation and institutional requirements. The patients/participants provided their written informed consent to participate in this study.

## Author contributions

SS prepared, edited, and submitted the manuscript. AY implemented the statistical analysis. BA, TN, RS, and CR provided corrections to statistical analysis and contributions to manuscript development. All authors contributed to the article and approved the submitted version.

## Conflict of interest

The authors declare that the research was conducted in the absence of any commercial or financial relationships that could be construed as a potential conflict of interest.

## Publisher's note

All claims expressed in this article are solely those of the authors and do not necessarily represent those of their affiliated organizations, or those of the publisher, the editors and the reviewers. Any product that may be evaluated in this article, or claim that may be made by its manufacturer, is not guaranteed or endorsed by the publisher.
